# Clinical Characteristics, Genetic Findings and Arrhythmic Outcomes of Patients with Catecholaminergic Polymorphic Ventricular Tachycardia from China: A Systematic Review

**DOI:** 10.3390/life12081104

**Published:** 2022-07-22

**Authors:** Justin Leung, Sharen Lee, Jiandong Zhou, Kamalan Jeevaratnam, Ishan Lakhani, Danny Radford, Emma Coakley-Youngs, Levent Pay, Göksel Çinier, Meltem Altinsoy, Amir Hossein Behnoush, Elham Mahmoudi, Paweł T. Matusik, George Bazoukis, Sebastian Garcia-Zamora, Shaoying Zeng, Ziliang Chen, Yunlong Xia, Tong Liu, Gary Tse

**Affiliations:** 1 Cardiac Electrophysiology Unit, Cardiovascular Analytics Group, China-UK Collaboration, Hong Kong, China; 1155108824@link.cuhk.edu.hk (J.L.); sharen212@link.cuhk.edu.hk (S.L.); 1155092270@link.cuhk.edu.hk (I.L.); 2 School of Data Science, City University of Hong Kong, Hong Kong, China; jiandong.zhou@ndm.ox.ac.uk; 3 Faculty of Health and Medical Sciences, University of Surrey, Guildford GU2 7XH, UK; k.jeevaratnam@surrey.ac.uk; 4 Kent and Medway Medical School, Canterbury CT2 7FS, UK; d.radford415@kmms.ac.uk (D.R.); e.coakleyyoungs72@kmms.ac.uk (E.C.-Y.); 5 Department of Cardiology, Dr Siyami Ersek Thoracic and Cardiovascular Surgery Training and Research Hospital, Istanbul 34147, Turkey; leventpay@hotmail.com (L.P.); cinierg@gmail.com (G.Ç.); 6 Department of Cardiology, University of Health Sciences, Diskapi Yildirim Beyazit Training and Research Hospital, Ankara 06145, Turkey; meltemaltinsoy@hotmail.com; 7 Universal Scientific Education and Research Network (USERN), Tehran University of Medical Sciences, Tehran 1416643931, Iran; ah-behnoush@student.tums.ac.ir (A.H.B.); e-mahmoudi@alumnus.tums.ac.ir (E.M.); 8 Department of Electrocardiology, Institute of Cardiology, Jagiellonian University Medical College, John Paul II Hospital, 31-202 Kraków, Poland; pawel.matusik@wp.eu; 9 Department of Cardiology, Larnaca General Hospital, Larnaca 6301, Cyprus; gbazoykis@med.uoa.gr; 10 Medical School, University of Nicosia, Nicosia 2408, Cyprus; 11 Cardiac Intensive Care Unit, Department of Cardiology, Delta Clinic, Rosario S2000, Argentina; szamora@sanatoriodelta.com.ar; 12 Department of Pediatric Cardiology, Guangdong Cardiovascular Institute, Guangdong Provincial Key Laboratory of South China Structural Heart Disease, Guangdong Provincial People’s Hospital, Guangdong Academy of Medical Sciences, Guangzhou 510080, China; doctorzsy@163.com; 13 Tianjin Key Laboratory of Ionic-Molecular Function of Cardiovascular Disease, Department of Cardiology, Tianjin Institute of Cardiology, Second Hospital of Tianjin Medical University, Tianjin 300211, China; chenziliang@tmu.edu.cn; 14 Department of Cardiology, First Affiliated Hospital of Dalian Medical University, Dalian 116014, China; yunlong_xia@126.com

**Keywords:** CPVT, *RyR2*, catecholaminergic polymorphic ventricular tachycardia

## Abstract

Introduction: Catecholaminergic polymorphic ventricular tachycardia (CPVT) is a rare inherited cardiac ion channelopathy. The present study aims to examine the clinical characteristics, genetic basis, and arrhythmic outcomes of CPVT patients from China to elucidate the difference between CPVT patients in Asia and Western countries. Methods: PubMed and Embase were systematically searched for case reports or series reporting on CPVT patients from China until 19 February 2022 using the keyword: “Catecholaminergic Polymorphic Ventricular Tachycardia” or “CPVT”, with the location limited to: “China” or “Hong Kong” or “Macau” in Embase, with no language or publication-type restriction. Articles that did not state a definite diagnosis of CPVT and articles with duplicate cases found in larger cohorts were excluded. All the included publications in this review were critically appraised based on the Joanna Briggs Institute Critical Appraisal Checklist. Clinical characteristics, genetic findings, and the primary outcome of spontaneous ventricular tachycardia/ventricular fibrillation (VT/VF) were analyzed. Results: A total of 58 unique cases from 15 studies (median presentation age: 8 (5.0–11.8) years old) were included. All patients, except one, presented at or before 19 years of age. There were 56 patients (96.6%) who were initially symptomatic. Premature ventricular complexes (PVCs) were present in 44 out of 51 patients (86.3%) and VT in 52 out of 58 patients (89.7%). Genetic tests were performed on 54 patients (93.1%) with a yield of 87%. RyR2, CASQ2, TERCL, and SCN10A mutations were found in 35 (71.4%), 12 (24.5%), 1 (0.02%) patient, and 1 patient (0.02%), respectively. There were 54 patients who were treated with beta-blockers, 8 received flecainide, 5 received amiodarone, 2 received verapamil and 2 received propafenone. Sympathectomy (*n* = 10), implantable cardioverter-defibrillator implantation (*n* = 8) and ablation (*n* = 1) were performed. On follow-up, 13 patients developed VT/VF. Conclusion: This was the first systematic review of CPVT patients from China. Most patients had symptoms on initial presentation, with syncope as the presenting complaint. RyR2 mutation accounts for more than half of the CPVT cases, followed by CASQ2, TERCL and SCN10A mutations.

## 1. Introduction

Cardiac ion channelopathies are hereditary conditions that increase a patient’s risk of developing spontaneous ventricular tachycardia/fibrillation (VT/VF) and sudden cardiac death (SCD) in the absence of structural heart disease [1,2,3,4,5,6]. Amongst different cardiac channelopathies, such as Brugada syndrome (BrS) and long QT syndrome, catecholaminergic polymorphic ventricular tachycardia (CPVT) is a rare, but lethal, condition that is less prevalent in Asia with an estimated global prevalence of 1 in 10,000 [7,8,9]. It is typically caused by mutations in either the ryanodine receptor 2 (RyR2) gene [10] or the calsequestrin 2 (CASQ2) gene [11,12], but mutations in other genes such as calmodulin (CALM) have been implicated in recent studies [13,14,15]. Patients with CPVT usually present in the first two decades of life, with symptoms of syncope or SCD manifested after physical or emotional distress, resulting in bidirectional VT [16]. In terms of patient management, although there is some evidence supporting the use of flecainide, given its RyR2 blocking properties [17], use of β-blockers without sympathomimetic activity remains the mainstay of treatment. Amongst patients who survived an attempt of SCD, or present with syncope/sustained VT/VF despite maximal medical therapy, current evidence recommends the use of an implantable cardioverter-defibrillator (ICD) for primary/secondary SCD prevention [18,19,20]. However, although ICD implantation is associated with reduced mortality amongst high risk CPVT patients, it should be noted that ICD shocks come with a risk of triggering ventricular tachyarrhythmia under a vicious cycle of adrenergic stimulation, thus potentially increasing the morbidity and mortality amongst this cohort of patients [21].

Globally, Western countries have mainly contributed to population-based data on CPVT. To the best of our knowledge, the largest registry reported 237 CPVT patients, which was created by the Pediatric and Congenital Electrophysiology Society of the United States [22,23]. In another multinational study, 101 CPVT patients who mainly came from France were reported [24], complementing smaller registry and case series studies by the same group [25,26]. In another study, 21 CPVT patients with CALM gene mutations were reported [13]. In contrast, limited data from Asia have been studied, which may be secondary to the lower prevalence of CPVT in Asia. Taking Japan as an example, 78 patients with CPVT were enrolled in a multi-centre registry [27]. Other studies from Japan identified 30 [28] and 29 patients [29] with CPVT, respectively. However, to date, no nation-wide registry has been established in China, and descriptions of CPVT patients have been limited to case reports or case series [12,30,31,32]. Moreover, as many of these studies were written in Chinese, researchers beyond China may have limited access to the studies due to the language barriers, which may have hindered researchers from gaining insights into the presentation and management of CPVT in China. Recently, our team conducted a situation analysis of the local CPVT burden in Hong Kong through a systematic literature search [33]. The present study, which is an extension of our previous work, has the aim of synthesizing evidence on the clinical characteristics, genetic basis, and arrhythmic outcomes of CPVT patients from China to examine the difference between CPVT patients in Asia and Western countries.

## 2. Methods

This systematic review was conducted in accordance with the Preferred Reporting Items for Systematic Reviews and Meta-Analyses (PRISMA) 2020 checklist [34].

### 2.1. Search Strategy and Selection Process

PubMed and Embase were systematically searched for case reports or case series that described CPVT patients from China until 19 February 2022, which allowed a primary synthesis of cases for analysis. Keywords of the search included: “Catecholaminergic Polymorphic Ventricular Tachycardia” or “CPVT”, with the country of author being limited to: “China” or “Hong Kong” or “Macau” in Embase, with no language or publication-type restriction. Case reports or case series that described patients with characteristics suggestive, but without a definite diagnosis of CPVT, were excluded. The diagnosis of CPVT was established based on the exercise treadmill test, adrenaline challenge test, or genetic testing as defined by the individual articles.

The articles that were identified from the databases were first screened by six reviewers (DR, MA, LP, ECY, JL, GT) according to their titles and abstracts. The full text of the articles was then assessed for eligibility based on the inclusion and exclusion criteria as stated above. Where overlapping cohorts were described, data were extracted from the publication with the largest cohort.

All articles that were finally included in this systematic review were critically appraised by two independent reviewers (JL, SL) based on the Joanna Briggs Institute Critical Appraisal Checklist to evaluate the information provided and the extent of bias that may have arisen in the articles [35].

### 2.2. Data Extraction and Statistical Analysis

The following clinical data were extracted by two reviewers (JL, GT) from the published studies: demographics: (1) sex, (2) age of presentation, (3) age of diagnosis, (4) family history of SCD or CPVT; initial presenting symptoms: (1) syncope, (2) palpitations, (3) chest pain, (4) seizures; electrocardiographic: the presence of (1) premature ventricular complexes (PVCs) or (2) VT/VF detected on electrocardiography, 24 h Holter studies or exercise stress testing, (3) the presence of bradycardic complications, (4) the presence of arrhythmias other than premature ventricular complexes (PVCs) /VT/VF; genetic: (1) the performance of genetic testing, (2) the method of genetic testing, (3) the results of genetic testing, (4) the interpretation of the variants found; other investigations: (1) the performance of electrophysiological study (EPS) and their respective results; (2) the performance of an echocardiogram and its results; (3) cardiac magnetic resonance imaging and results; treatment: (1) the prescription of anti-arrhythmic pharmacological agents, (2) the performance of sympathectomy (3) the implantation of ICD (4) the performance of ablation. Categorical variables were summarized as frequency (%) and continuous variables were expressed as median (Q1–Q3). The present study was conducted in accordance with the Preferred Reporting Items for Systematic Reviews and Meta-Analyses (PRISMA) 2020 guideline. Supplementary Table S1 summarizes the features of the present study that fulfills the PRISMA checklist. All statistical analysis was performed using International Business Machines (IBM) Statistical Product and Service Solutions (SPSS) Statistics 27.

The individual cases were also analyzed according to the diagnostic criteria proposed by the 2013 Heart Rhythm Society (HRS)/European Heart Rhythm Association (EHRA)/Asia-Pacific Heart Rhythm Society (APHRS) expert consensus statement (Supplementary Table S2) [9], evaluating whether the cases described in the included articles have fulfilled one or more of the criteria stated. All genetic variants described in the studies were searched for within the genetic database ClinVar to determine their possible pathogenicity and novelty, and within VARSOME for further prediction. Mutation hotspots for RyR2 were also identified according to the criteria set by Priori and Chen et al. [36]. For reporting, the following terminology recommended by the American College of Medical Genetics (ACMG) was used: “pathogenic”, “likely pathogenic”, “uncertain significance”, “likely benign”, and “benign” [37].

## 3. Results

### 3.1. Study Selection

**Figure 1:**
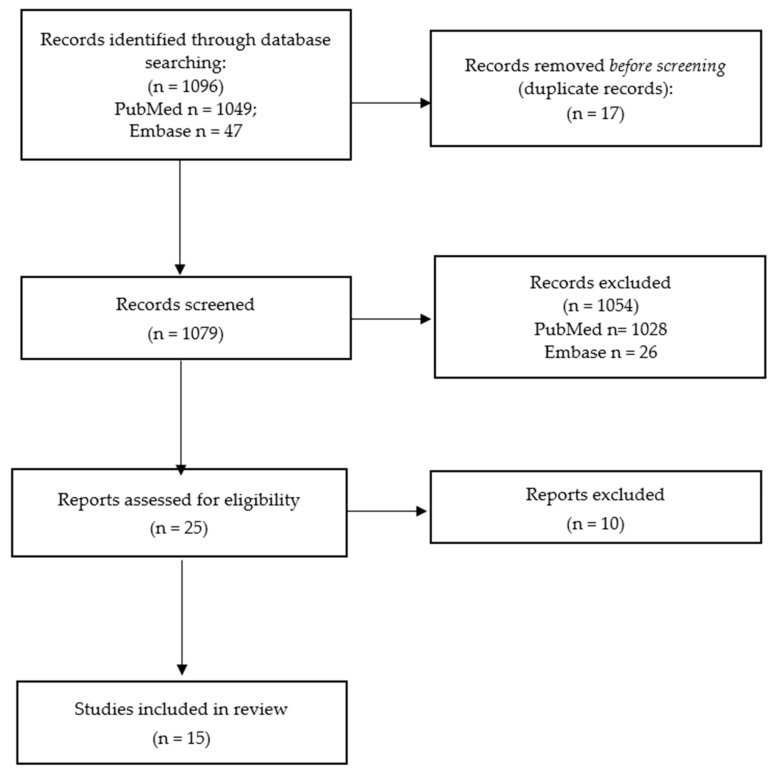
PRISMA 2020 flow diagram for systematic review.

The selection process of articles to be included in this systematic review is summarized by the PRISMA 2020 flow diagram (Figure 1).

A systematic search of the PubMed and Embase databases yielded 1049 and 47 articles, respectively. After the exclusion of duplicated articles found in both databases, 1079 articles were included for the initial screening by reviewing the title and abstracts, where 25 articles were found to be eligible for the full text assessment. After the elimination of articles that fell under the exclusion criteria, did not meet the inclusion criteria, and articles that described overlapping cohorts [30,32,38,39], a total of 58 unique cases with a definite diagnosis of CPVT from eight cities in China from 15 studies were included [12,31,40,41,42,43,44,45,46,47,48,49,50,51,52]. All articles were critically appraised according to the Joanna Briggs Institute Critical Appraisal Checklist and were considered to be suitable for inclusion.

### 3.2. Baseline Characteristics and Predictors

CPVT cases were reported in the following cities: Beijing (*n* = 22) [12,40,41,42], Hong Kong (*n* = 16) [31], Guangzhou (*n* = 7) [43,48], Nanjing (*n* = 6) [44], Shanghai (*n* = 4) [45,46,49,51], Chengdu (*n* = 1) [47], Shenzhen (*n* = 1) [50] and Xi’an (*n* = 1) [52]. Their clinical characteristics and test results are shown in Table 1. A total of 21 patients fulfilled at least two criteria and 41 patients fulfilled one criterion of the 2013 Heart Rhythm Society (HRS)/European Heart Rhythm Association (EHRA)/Asia Pacific Heart Rhythm Society (APHRS) expert consensus statement (Supplementary Table S2). A total of 22 (37.9%) patients were female and all patients were of Han Chinese origin. All patients except one presented at or before 19 years of age. The median age of presentation and diagnosis was 8.0 (5.0–11.8) and 10.1 (8.3–13.0) years old, respectively, with a median delay of 16 (3.0–46.8) months. A total of 56 patients (96.6%) were initially symptomatic. PVCs were present in 44 out of 51 patients and VT was present in 52 out of 58 patients. An abnormal echocardiogram was found in four patients. Duan et al. reported a patient with mild mitral and tricuspid regurgitation with a subsequent finding of a mildly dilated left ventricle on follow-up [47]. Ge et al. reported a patient with a mildly dilated left ventricle [40]. Lin et al. reported a patient with a thinner apical myocardium of the left ventricle [48]. Abnormality on echocardiogram was also found in a patient in a study conducted by Lee et al. but findings were not reported in the study [31]. Genetic tests were performed on 54 patients (93.1%) with a yield of 87%. RyR2, CASQ2, TERCL, and SCN10A mutations were found in 35 (71.4%), 12 (24.5%), one (0.02%) patient, and one patient (0.02%), respectively (Table 2). 

Pharmacological and non-pharmacological treatments for this cohort are summarized in Table 3. In terms of pharmacological treatment, 54 patients were prescribed β-blocker, eight patients received flecainide, five patients received amiodarone, two received verapamil and two received propafenone. On the other hand, in terms of interventional treatment, sympathectomy (*n* = 10), ICD implantation (*n* = 8), and ablation (*n* = 1) were performed. On follow-up, 13 patients developed incident VT/VF. 

## 4. Discussion

To the best of our knowledge, the present study is the first systematic review of published case reports/series of CPVT patients from China. The main findings are that: (1) RyR2 mutations account for over half of the CPVT cases, (2) 24 RyR2 variants, eight CASQ2 variants, two TERCL variants and one SCN10A variant were described, (3) β-blockers are used in 93.1% of the cases, followed less frequently by flecainide, amiodarone, verapamil and propafenone, and (4) 17.2% patients underwent cardiac sympathectomy, 13.8% received ICDs and 1.7% received ablation.

### 4.1. Demographics of Catecholaminergic Polymorphic Ventricular Tachycardia in China 

In this systematic review, CPVT cases were reported mostly in major cities such as Beijing and Hong Kong. This may be due to the fact that these cities have better human and technical resources which allow easier detection of such rare cardiac ion channelopathy. In contrast, cases in rural areas may be underreported, thus the true demographics of CPVT in China may not be assessed. This highlights the need for a national registry for better documentation of the disease, and to allow further studies to comprehensively explore the prevalence as well as any predominant genetic variant in different parts of China.

### 4.2. Clinical Characteristics and Risk Stratification in Catecholaminergic Polymorphic Ventricular Tachycardia

SCD is an important clinical problem globally, with congenital and acquired causes [65,66,67,68]. Regarding the phenotype of congenital cardiac ion channelopathies, CPVT is characterized by sustained polymorphic VT under adrenergic stimulation, which can be induced by physical or emotional stress. The symptoms include palpitation, syncope and episodes of SCD, often occurring in the early decades of the patient’s life [19]. Similar to BrS, this hereditary cardiac ion channelopathy is dynamic and can show normal electrocardiograms at baseline. Additionally, there is also incomplete penetrance with variable expression, which makes disease diagnosis even more challenging despite the presence of SCD history amongst one-third of the patient families [69]. Significant delays from the date of initial presentation to the date of diagnosis of around six months have been reported in international registry studies on North American and European patients [23,70]. In contrast, the epidemiology and characteristics of studies in Asia are limited. In China, cases of CPVT have been limited to small case reports or case series on specific genetic mutations. For example, a small-scale cohort study has examined pediatric CPVT patients with CASQ2 variants in China [12]. However, the limited cohort size prevents the systematic analysis of patient characteristics and treatment management. 

The incidence of adverse outcomes in CPVT patients has been examined in multiple studies, which have focused on syncopal events and SCD [24,25,71,72]. Earlier evidence suggests that subjects who are initially symptomatic, who constituted the majority of the cohort represented in this systematic review, as well as those who are younger at diagnosis with no administration of β-blocker therapy, have a significantly higher susceptibility to cardiac events, including syncope, aborted cardiac arrest, and/or SCD [24]. However, the concept was challenged by a recent international pediatric CPVT cohort study, which demonstrated that the age of symptom onset and sex were not independent predictors of the time to the first arrhythmic event [73]. Instead, proband status remains to be the critical determinant for the time to cardiac event occurrence. The findings from this study highlight the need for further research on the SCD risk stratification of CPVT patients, in addition to the essential role of genetic diagnosis in this patient cohort.

On follow-up of the CPVT patients in this systematic review, most patients had not reported any further symptoms after the use of pharmacological therapy and interventional treatment if necessary. However, 21.6% had experienced cardiac events, in which three patients had SCD due to medication non-compliance or emotional stress. This finding highlights the need for CPVT patients to have good compliance with taking medications and have better control over emotional stress, echoing suggestions proposed by previous studies [61,74]. Further studies should also be conducted to learn the long-term prognosis of these patients following initiation of treatment, as the follow-up duration of the included studies was heterogeneous and thus might not provide a comprehensive picture. 

Regarding electrocardiographic parameters, their effectiveness in the prognostication for VT/VF in patients with CPVT remains uncertain due to a relative shortage of literature assessing such aspects. The challenge in SCD risk stratification is further worsened by the dynamic electrical nature of the condition with the maintenance of normal resting electrocardiograms [19]. In a pluripotent stem cell-derived human cardiomyocyte model, it was demonstrated that the arrhythmic substrate was only unmasked under the catecholamine-induced phosphorylation of the ryanodine receptor due to an increase in spatiotemporal dispersion of calcium ions during diastole, which explained the lack of arrhythmic activity in the absence of stress [75]. In the assessment of SCD as a disease outcome, some studies have demonstrated significant differences in QRS durations of the recorded PVCs between patients who remained alive and those who suffered SCD during follow-up, whereas most of the other ECG variables, such as heart rate and QTc interval, failed to demonstrate any notable variations with time [24]. These findings can be attributed to distinct cellular mechanisms underlying arrhythmogenesis in CPVT compared to other ion channelopathies. In CPVT, the predominant abnormality involves defective calcium release channels leading to calcium-driven arrhythmias with conduction and repolarization defects being secondary [76,77]. Moreover, the dynamic nature of the disease means that ECG indices may be normal at rest and not distinguishable from normal values, even though early repolarization has been reported as being an arrhythmic risk indicator [78,79]. In contrast, in BrS or LQTS, the affected channels are predominantly involved in conduction and repolarization [80,81], which could explain why ECG indices reflect that these physiological processes are predictive of arrhythmic events [82,83,84,85,86,87].

### 4.3. Genetic Variants Underlying Catecholaminergic Polymorphic Ventricular Tachycardia

In accordance with existing literature, RyR2 and CASQ2 remained the two most common mutated genes that underlie the development of CPVT. The present study identified 24 RyR2 variants. Of these, 13 have been reported outside China: c.490C > T [53], c.2410C > T (RCV000639160.2), c.6886G > A (RCV000465586.1), c.7202G > A [54], c.7258A > G [55], c.7420A > G [56], c.10046C > T [57,58], c.11836G > A [59,60], c.12272C>T (RCV00182811.1), c.12475C > A [61], c.13933T > C [62], c.14159T > C (RCV000182842.2), and c.14848G > A [60]. CASQ2, in comparison, accounts for a fewer proportion of CPVT cases. In our study, eight variants were reported. Three have been reported from publications arising from outside China: c.97C > T [63], c.98G > A [64], and c.748C > T (RCV000694480.2), with five novel mutations. 

Since both genes are key contributors to the control of calcium release from the sarcoplasmic reticulum, it has been hypothesized that the spontaneous release of calcium during diastole leads to delayed after-repolarization, thus subsequently triggering arrhythmic activity [88]. However, it should be noted that different mutations of the same gene could give rise to different cellular and molecular adaptive mechanisms, which may lead to different disease manifestations and affect treatment efficacy. A mice model comparing CASQ2, D307H, and R33Q mutants found that whilst the same phenotype was displayed, the two mutants varied in the extent of small Heat Shock Protein (HSP) phosphorylation, which protects against cardiomyocyte remodeling and thus reduces the arrhythmic risk [89]. Given that RyR2 variants have been reported in previous animal studies to be associated with a disruption in calcium homeostasis and a reduction in conduction velocity, functional studies should also be conducted to identify how such variants could lead to the generation of electrophysiological substrates [76,90,91]. Furthermore, the interaction between mutations amongst patients with multiple co-existing genetic variants also requires further elucidation. In the Pediatric and Congenital Electrophysiology Society (PACES) CPVT registry, 15 of the 193 patients who had undergone genetic testing were found to have at least two CPVT-related genetic mutations [22]. Whilst the cumulative gene dosage phenomenon was found to increase the risk of SCD amongst patients with other hereditary cardiac arrhythmic syndromes such as arrhythmogenic right ventricular cardiomyopathy (ARVC) and long QT syndrome, further studies are needed to provide evidence for such phenomenon amongst patients with CPVT [89,92]. 

With the increased use of genetic screening in families with a history of SCD, there is increasing evidence of the involvement of different genes in the development of CPVT. Recently, a panel of gene curation experts reviewed all published evidence for CPVT-associated genes curated by three independent genes, and identified five more genes with concrete evidence for their causation of CPVT, aside from RyR2 and CASQ2 [93]. RYR2, CALM1, CALM2, and CALM3 were inherited in an autosomal dominant inheritance, whilst CASQ2, TRDN, and TECRL had an autosomal recessive inheritance [93]. In the present study, mutations in genes beyond RyR2 and CASQ2 were also found, which is consistent with the recent literature. Two TERCL variants as well as one SCN10A variant, both of which are novel in nature, were reported. Mutations of the TERCL have recently been reported in a family with stress-induced ventricular arrhythmias [94]. Since TERCL is another critical player in the maintenance of intracellular calcium hemostasis within myocardial cells, the pathogenic mechanism of the TERCL mutants is likely to be similar to the RyR2 and CASQ2 mutants [94]. 

In contrast, although the expert panel did not identify SCN10A as a causative mutation of CPVT, SCN10A variants were reported in patients with multiple mutations, in addition to patients with BrS [52,95]. Basic studies have also shown that SCN10A variants affect the myocardial sodium current and therefore affect the arrhythmogenic susceptibility of the patient [96,97]. As the SCN10A variant has not been previously detected in a CPVT patient, further study is needed to elucidate the complex causative relationship between genetic mutations and the CPVT phenotype. Although there is insufficient evidence to show that there is an epidemiological predilection between different CPVT-associated mutations, the lack of reports on CALM1, CALM2, CALM3, and TRDN mutations highlights their rarity and the need to examine the global trends of genetic mutations in CPVT.

### 4.4. Strengths and Limitations

There are several major strengths of the present study. First of all, to the best of our knowledge, the present study is the first systematic summary of published CPVT cases across all ages in China, which presented a comprehensive analysis of clinical characteristics, genetic basis, and arrhythmic outcomes of CPVT patients from China. Since China accounts for a large population in Asia, the examination of CPVT patients in China provides insights into clinical, genetic and epidemiological differences between Asian and Western CPVT patients. By including a comprehensive extraction and integration of data, this study allows easier interpretation of CPVT cases in China by researchers beyond China, which can help to provide a critical piece of a missing puzzle in the analysis of the global epidemiology of CPVT. In addition, the present study also forms a basis for further studies in comparing CPVT between China and other Asian or non-Asian populations. With a focus on the genetic mutations reported in the published cases, the present study provides further evidence to support the disease causation by genetic mutations beyond the RyR2 and CASQ2 genes.

However, a number of limitations should also be noted with caution when interpreting the findings from the present study. Firstly, all data were extracted from case reports or case series. Given the nature of the study, the data could not be used for prognostication given the inherent selection bias for patients who were more severely affected and had a need for hospital admission. Thus, the prognostic value of the genetic variants, in addition to other clinical and epidemiological factors, was not evaluated. Furthermore, as a systematic review of published literature, the case details could only be extracted online from the publications. The present study was unable to access the primary data source from hospitals in China to fill in the missing details from the publications, which limited the inter-publication consistency and comprehensiveness of the extracted data. Additionally, the absence of an established national registry in China also led to the inability to include all the domains that were assessed in this current study for all studies, therefore the data may not reflect the actual picture of CPVT patients from China, especially regarding arrhythmic events upon follow-up. The extent of the patient details that were documented inevitably varied between different articles, which limited the analysis that could be done in the present study. The change in diagnostic and management guidelines over time also contributed to the inconsistency of information provided, particularly in terms of genetic testing. The availability of next-generation genome sequencing (NGS) allows the identification of many more novel mutations, which may not be identified amongst patients in the past. Finally, the pattern of healthcare resource utilization of CPVT patients was recently examined in a local cohort [98], however they have not been systematically evaluated for the wider China area and a coordinated effort is required in the future to achieve this.

## 5. Conclusions

To conclude, this is the first systemic review of CPVT patients from China. Most patients had symptoms on initial presentation, with syncope as the presenting complaint. Similar to patients in the Western world, RyR2 mutation accounts for more than half of the CPVT cases, followed by CASQ2, TERCL, and SCN10A mutations. Further study is needed to elucidate the causative relationship between the genetic mutations and the disease phenotype to bring about improvements in the prognostication of CPVT patients.

## Figures and Tables

**Table 1:** Baseline clinical and demographic characteristics of CPVT patients from China.

Parameter	Median (Q1–Q3)/Frequency (%)
Female	22 (37.9)
Presentation Age (years)	8.0 (5.0–11.8)
Diagnosis Age (years)	10.1 (8.3–13.0)
Presentation to Diagnosis (months)	16 (3.0–46.8)
Family History of CPVT/SCD	14 (24.1)
Initially symptomatic	56 (96.6)
Initial syncope	54 (93.1)
Initial VT/VF/SCD	15 (25.9)
Initial palpitations	12 (20.7)
Initial chest pain	7 (12.1)
Initial seizure	17 (29.3)
PVC	44 (75.9)
VT/VF	52 (89.7)
VT/VF post-presentation	13 (22.4)
Echocardiogram	43 (74.1)
Abnormal echocardiogram	4 (9.3)
Cardiac MRI performed	5 (8.6)
Abnormal cardiac MRI	0 (0)
Genetic Test	54 (93.1)
Positive Genetic Test	47 (87)
Adrenaline Challenge	9 (15.5)
Positive Adrenaline Challenge	9 (100)
Exercise Tolerance Test	46 (79.3)
Positive Exercise Tolerance Test	44 (97.8)
EPS	3 (5.2)
Positive EPS	3 (100)
Holter Study	43 (74.1)
Arrhythmia in Holter Study	34 (81)

CPVT: catecholaminergic polymorphic ventricular tachycardia; SCD: sudden cardiac death; VT: ventricular tachycardia; VF: ventricular fibrillation; PVC: premature ventricular complex; MRI: magnetic resonance imaging; EPS: electrophysiological study.

**Table 2:** (a). Genetic test results (RyR2). (b) Genetic test results (non-RyR2).

(a)									
**Gene**	**Mutation**	**Region in Genome**	**Coding Effect**	**Mutation Type**	**Mutation Hotspots**	**Pathogenicity**	**Prediction**	**Reported Mutation** **Outside China**	**Reference**
*RyR2*	c.229C > T	Exon 3	P77S	Substitution	Domain I	Uncertain significance	Uncertain significance	Novel mutation	Ge 2017 [40]
*RyR2*	c.490C > T	Exon 8	P164S	Substitution	Domain I	Uncertain significance	Likely Pathogenic	No: [53]	Lin 2018 [48]
*RyR2*	c.494C > A	Exon 8	A165D	Substitution	Domain I	Uncertain significance	Pathogenic	Novel mutation	Xiong 2018 [51]
*RyR2*	c.1639A > C	Exon 17	N547H	Substitution	Non-hotspot	Uncertain significance	Uncertain significance	Novel mutation	Ge 2017 [40]
*RyR2*	c.2410C > T	Exon 22	L804F	Substitution	Non-hotspot	Likely benign	Benign	RCV000639160.2	Ge 2017 [40]
*RyR2*	c.5564C > A	Exon 37	A1855D	Substitution	Non-hotspot	Uncertain significance	Uncertain significance	Novel mutation	Zhang 2019 [52]
*RyR2*	c.6577G > T	Exon 43	V2193L	Substitution	Non-hotspot	Uncertain significance	Uncertain significance	Novel mutation	She 2020 [50]
*RyR2*	c.6886G > A	Exon 45	E2296K	Substitution	Domain II	Uncertain significance	Uncertain significance	RCV000465586.1	Hou 2019 [49]
*RyR2*	c.7202G > A	Exon 47	R2401H	Substitution	Domain II	Likely Pathogenic	Pathogenic	No: [54]	Lee 2021 [31]
*RyR2*	c.7258A > G	Exon 48	R2420G	Substitution	Domain II	Uncertain significance	Uncertain significance	No: [55]	Ge 2017 [40]
*RyR2*	c.7420A > G	Exon 49	R2474G	Substitution	Domain II	Uncertain significance	Likely Pathogenic	No: [56]	Lee 2021 [31]
*RyR2*	c.7580T > G	Exon 50	L2527W	Substitution	Domain II	Uncertain significance	Uncertain significance	Novel mutation	Duan 2018 [47]
*RyR2*	c.10046C > T	Exon 69	S3349L	Substitution	Non-hotspot	Uncertain significance	Uncertain significance	No: [57,58]	Lee 2021 [31]
*RyR2*	c.11836G > A	Exon 88	G3946S	Substitution	Domain III	Pathogenic	Pathogenic	No: [59,60]	Ge 2017, Lee 2021 [31,40]
*RyR2*	c.12014A > T	Exon 90	E4005V	Substitution	Domain III	Uncertain significance	Uncertain significance	Novel mutation	Yang 2021 [42]
*RyR2*	c.12272C > T	Exon 90	A4091V	Substitution	Domain III	Uncertain significance	Uncertain significance	RCV00182811.1	Yang 2021 [42]
*RyR2*	c.12475C > A	Exon 90	Q4159K	Substitution	Domain III	Uncertain significance	Likely Pathogenic	No: [61]	Lee 2021 [31]
*RyR2*	c.13933T > C	Exon 96	W4645R	Substitution	Domain IV	Uncertain significance	Uncertain significance	No: [62]	Ge 2017 [40]
*RyR2*	c.14159T > C	Exon 99	L4720P	Substitution	Domain IV	Uncertain significance	Uncertain significance	RCV000182842.2	Lee 2021 [31]
*RyR2*	c.14570T > G	Exon 101	I4857S	Substitution	Domain IV	Uncertain significance	Uncertain significance	Novel mutation	Ge 2017 [40]
*RyR2*	c.14593C > A	Exon 102	L4865I	Substitution	Domain IV	Uncertain significance	Uncertain significance	Novel mutation	Ge 2017 [40]
*RyR2*	c.14834A > G	Exon 105	Q4945R	Substitution	Domain IV	Likely benign	Uncertain significance	Novel mutation	Ge 2017 [40]
*RyR2*	c.14848G > A	Exon 105	E4950K	Substitution	Domain IV	Uncertain significance	Likely Pathogenic	No: [60]	Lee 2021 [31]
*RyR2*	c.14861C > G	Exon 105	A4954G	Substitution	Domain IV	Uncertain significance	Uncertain significance	Novel mutation	Lee 2021 [31]
(b)									
**Gene**	**Mutation**	**Region in Genome**	**Coding Effect**	**Mutation Type**		**Pathogenicity**	**Prediction**	**Reported Mutation** **Outside China**	**Reference**
*CASQ2*	c.97C > T	Exon 1	R33X	Substitution		Likely Pathogenic	Pathogenic	No: [63]	Gao 2018, Li Q 2019 [12,41]
*CASQ2*	c.98G > A	Exon 1	R33Q	Substitution		Uncertain significance	Uncertain significance	No: [64]	Li Q 2019 [12]
*CASQ2*	c.244C > T	Exon 1	Q82X	Substitution		Uncertain significance	Pathogenic	Novel mutation	Ge 2017 [40]
*CASQ2*	c.532+1G > A	IVS		Splice site mutation		Uncertain significance	Pathogenic	Novel mutation	Li Q 2019 [12]
*CASQ2*	c.748C > T	Exon 7	R250C	Substitution		Uncertain significance	Uncertain significance	RCV000694480.2	Gao 2018, Li Q 2019 [12,40]
*CASQ2*	c.838 + 1G > A	IVS		Splice site mutation		Uncertain significance	Pathogenic	Novel mutation	Li Q 2019 [12]
*CASQ2*	c.1074_1075delinsC	Exon 11	E359Rfs*12	Deletion and insertion		Uncertain significance	Pathogenic	Novel mutation	Li Q 2019 [12]
*CASQ2*	c.1175_1178delACAG	Exon 11	D392Vfs*84	Deletion		Uncertain significance	Pathogenic	Novel mutation	Li Q 2019 [12]
*TECRL*	c.587C > T	Exon 6	R196Q	Substitution		Uncertain significance	Uncertain significance	Novel mutation	Xie 2019 [46]
*TECRL*	c.918+3T > G	IVS		Splice site mutation		Uncertain significance	Uncertain significance	Novel mutation	Xie 2019 [46]
*SCN10A*	c.4086G > C	Exon 22	Q1362H	Substitution		Uncertain significance	Uncertain significance	Novel mutation	Zhang 2019 [52]

**Table 3:** Management for CPVT patients in China.

Treatment	Frequency (%)
β-blocker	54 (93.1)
Verapamil	2 (3.4)
Amiodarone	5 (8.6)
Flecainide	8 (13.8)
Propafenone	2 (3.4)
Sympathectomy	10 (17.2)
ICD implantation	8 (13.8)
Ablation	1 (1.7)

## Data Availability

The data described in this study are available from the published studies.

## Supplementary Materials

The following supporting information can be downloaded at: https://www.mdpi.com/article/10.3390/life12081104/s1. Table S1: PRISMA checklist; Table S2: Diagnostic Criteria.
