# Supplementary material for: Clinical Characteristics, Genetic Findings and Arrhythmic Outcomes of Patients with Catecholaminergic Polymorphic Ventricular Tachycardia from China: A Systematic Review
Source: Life (Basel). 2022 Jul 22;12(8):1104. doi: 10.3390/life12081104 (PMC9330865; doi:10.3390/life12081104)
Supplement: Supplementary file 1 [file life-12-01104-s001.zip › CPVT Supplementary Table S2 (Diagnostic Criteria).pdf]

Supplementary Table S2. Details on the diagnostic criteria met for individual CPVT patients. The criteria proposed by the 2013 HRS/EHRA/APHRS expert consensus statement were used:

1. CPVT is diagnosed in the presence of a structurally normal heart, normal ECG, and unexplained exercise or catecholamine-induced bidirectional VT, polymorphic ventricular premature beats or VT in individuals <40 years of age.
2. CPVT is diagnosed in patients (index case or family member) who have a pathogenic mutation.
3. CPVT is diagnosed in family members of a CPVT index case with a normal heart who manifests exercise-induced PVCs or bidirectional/polymorphic VT.
4. CPVT can be diagnosed in the presence of a structurally normal heart and coronary arteries, normal ECG, and unexplained exercise or catecholamine-induced bidirectional VT, polymorphic ventricular premature beats or VT in individuals >40 years of age.

Case number	Criteria 1	Criteria 2	Criteria 3	Criteria 4
CNChen202001	1	- (no further detail on mutation was provided)	0	0
CNDuan201801	1	VUS	0	0
CNGao201801 (Proband)	1	1 (likely pathogenic)	0	0
CNGao201802 (Brother 1)	1	VUS	1	0
CNGao201803 (Brother 2)	1	VUS	1	0
CNGe201701	1	0 (likely benign)	0	0
CNGe201702	1	VUS	0	0
CNGe201703	1	1	0	0
CNGe201704	1	VUS	0	0
CNGe201705	0	0 (likely benign)	0	0
CNGe201706	0	VUS	0	0
CNGe201707	0	VUS	0	0
CNGe201708	1	VUS	0	0
CNGe201709	1	0	0	0
CNGe201710	1	VUS	0	0
CNGe201711	1	- (not done)	0	0
CNHou201901	1	VUS	0	0
CNLiQ201901 (Family 1)	1	1 (likely pathogenic)	0	0
CNLiQ201902 (Family 1)	1	1 (likely pathogenic)	1	0
CNLiQ201903 (Family 1)	1	1 (likely pathogenic)	1	0
CNLiQ201904 (Family 2)	1	VUS	0	0
CNLiQ201905 (Family 3)	1	VUS	0	0
CNLiQ201906 (Family 4)	1	VUS	0	0
CNLiZ201901	- (no details provided)	- (not done)	0	0
CNLiZ201902	- (no details provided)	- (no further detail on mutation was provided)	0	0

CNLiZ201903	- (no details provided)	- (no further detail on mutation was provided)	0	0
CNLiZ201904	- (no details provided)	- (no further detail on mutation was provided)	0	0
CNLiZ201905	- (no details provided)	- (no further detail on mutation was provided)	0	0
CNLin201802 (Sister)	0	VUS	- (proband with unknown diagnosis)	0
CNLin201805 (Father)	0	VUS	- (proband with unknown diagnosis)	1
CNShe202001	1	VUS	0	0
CNXie201901 (Proband)	1	VUS	0	0
CNXiong201801	1	VUS	0	0
CNYang202101	- (no details provided)	VUS	0	0
CNYang202102	- (no details provided)	VUS	0	0
CNZhang201901	1	VUS	0	0
CNZhao201201	0	- (no further detail on mutation was provided)	0	0
CNZhao201202	0	- (no further detail on mutation was provided)	0	0
CNZhao201203	0	- (no further detail on mutation was provided)	0	0
CNZhao201204	0	- (no further detail on mutation was provided)	0	0
CNZhao201205	0	- (no further detail on mutation was provided)	0	0
CNZhao201206	0	- (no further detail on mutation was provided)	0	0
CNLee202101	1	1	0	0
CNLee202102	1	1	0	0
CNLee202103	1	1	0	0
CNLee202104	1	1	0	0
CNLee202105	0	1	1 (brother, mother, maternal granduncle)	0
CNLee202106	1	1	1 (brother, mother – not related to CNLee202105)	0
CNLee202107	1	1	0	0
CNLee202108	1	1	0	0
CNLee202109	1	0	0	0
CNLee2021010	1	1	0	0
CNLee2021011	0	1	1 (father, sister)	0
CNLee2021012	1	1	0	0
CNLee2021013	1	0	0	0
CNLee2021014	1	1	0	0
CNLee2021015	1	1	0	0
CNLee2021016	1	1	0	0
